# Auto-amputation of an Entire Foot with Ankle in a Diabetic Patient

**DOI:** 10.22114/ajem.v0i0.245

**Published:** 2019-08-17

**Authors:** Utsav Anand Mani, Danish Ansari, Firdaus Behram Bhot, Elizabeth Sada, Rajesh Ursekar

**Affiliations:** Department of Emergency Medicine, Bharati Hospital, Maharashtra, India.

## Case presentation

**Figure 1:**
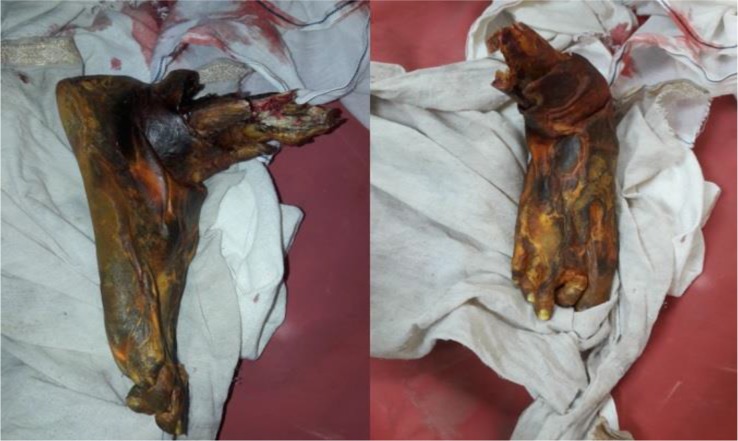
The dry, shrunken, mummified and black appearance of the limb is characteristic of dry gangrene that can be associated with diabetes; The yellowish discolouration is due to various home remedies tried on the patient.

A 62-year-old male came to emergency department (ED) with a complaint of “separation of foot” during sleep at night. He noticed he lost his foot while getting up from bed to go to toilet at midnight. Upon arrival in ED, his blood pressure was 218/96 mmHg and random blood sugar 556mg/dl. The patient had large ketone bodies in urine. He was a known case of diabetes and hypertension for many years. He was on homeopathic medications for his diabetes, hypertension and diabetic foot. He refused any form of invasive treatment and hospitalization. The patient was adamant on not getting admitted and wanted to continue his homeopathic medications as before. He only wanted his leg stump covered with a dressing after which he was discharged (figure 1).

## Learning points

Auto-amputation is the spontaneous falling of an appendage from the body due to long standing pathology. Although rare it has been reported in forms of case reports for digits, pinna, penis, appendix, breast, tongue and visceral tumours. Diabetes, Atherosclerosis, Ainhum, Pseudoainhum, vascular compromise in visceral tumours, dry gangrene, wet gangrene and gas gangrene are some of the many causes of auto-amputations (1, 2). In a case of a diabetic patient a combination of neuropathy, ulceration, microangiopathy, long standing metabolic derangement, repeated infections, deep abscesses, osteomyelitis ischaemia and neglect can push the limb towards a dry gangrene (3). Such a risk for a dry gangrene ending in auto-amputation in a diabetic patient is very high. An amputation in a diabetic foot maybe minor or major based on whether its proximal or distal to the metatarsal joint (4).
